# Identification of a Rare Exon 19 Skipping Mutation in ALMS1 Gene in Alström Syndrome Patients From Two Unrelated Saudi Families

**DOI:** 10.3389/fped.2021.652011

**Published:** 2021-04-26

**Authors:** Omar I. Saadah, Babajan Banaganapalli, Naglaa M. Kamal, Ahmed N. Sahly, Hadeel A. Alsufyani, Arif Mohammed, Aftab Ahmad, Khalidah Khalid Nasser, Jumana Y. Al-Aama, Noor Ahmad Shaik, Ramu Elango

**Affiliations:** ^1^Princess Al-Jawhara Center of Excellence in Research of Hereditary Disorders, King Abdulaziz University, Jeddah, Saudi Arabia; ^2^Pediatric Gastroenterology Unit, Department of Pediatrics, Faculty of Medicine, King Abdulaziz University, Jeddah, Saudi Arabia; ^3^Department of Genetic Medicine, Faculty of Medicine, King Abdulaziz University, Jeddah, Saudi Arabia; ^4^Department of Pediatrics, Al-Hada Armed Forces Hospital, Taif, Saudi Arabia; ^5^Pediatric Hepatology Unit, Department of Pediatrics, Faculty of Medicine, Cairo University, Cairo, Egypt; ^6^Department of Neurosciences, King Faisal Specialist Hospital and Research Centre, Jeddah, Saudi Arabia; ^7^Department of Physiology, Faculty of Medicine, King Abdulaziz University, Jeddah, Saudi Arabia; ^8^Department of Biology, College of Science, University of Jeddah, Jeddah, Saudi Arabia; ^9^Department of Health Information Technology, Faculty of Applied Studies, King Abdulaziz University, Jeddah, Saudi Arabia; ^10^Department of Medical Laboratory Technology, Faculty of Applied Medical Sciences, King Abdulaziz University, Jeddah, Saudi Arabia

**Keywords:** Alström syndrome, ALMS1, splice site mutation, rare variant, Saudi Arabia

## Abstract

**Background:** Alström syndrome (AS) is a very rare childhood disorder characterized by cardiomyopathy, progressive hearing loss and blindness. Inherited genetic variants of ALMS1 gene are the known molecular cause of this disease. The objective of this study was to characterize the genetic basis and understand the genotype–phenotype relationship in Saudi AS patients.

**Methods:** Clinical phenotyping and whole-exome sequencing (WES) analysis were performed on six AS patients belonging to two unrelated consanguineous Saudi families. Sanger sequencing was performed to determine the mode of inheritance of ALMS1 variant in first-degree family relatives and also to ensure its rare prevalence in 100 healthy population controls.

**Results:** We identified that Alström patients from both the families were sharing a very rare ALMS1, 3′-splice site acceptor (c.11873−2 A>T) variant, which skips entire exon-19 and shortens the protein by 80 amino acids. This disease variant was inherited by AS patients in autosomal recessive mode and is not yet reported in any population-specific genetic databases. AS patients carrying this mutation showed heterogeneity in clinical presentations. Computational analysis of the mutant centroid structure of ALMS1 mRNA revealed that exon-19 skipping enlarges the hairpin loop and decreases the free energy, eventually affecting its folding pattern, stability, and function. Hence, we propose c.11873–2A as an AS causative potential founder mutation in Saudi Arabia because it is found in two families lacking a common lineage.

**Conclusions:** We conclude that WES analysis potentially helps in clinical phenotyping, early diagnosis, and better clinical management of Alström patients showing variable clinical expressivity.

## Introduction

Alström syndrome (AS; OMIM 203800), first described in 1959, is an inherited ciliary disorder seen in early childhood. The affected children typically present a complex array of clinical characteristics including childhood obesity, early onset of blindness, neurosensory hearing loss, type 2 diabetes mellitus, progressive liver and kidney disease, hypertriglyceridemia, and cardiomyopathy (1). AS patients also present short stature, hypogonadotropic hypogonadism in affected males, and polycystic ovarian syndrome (PCOS) in affected females. In AS patients, clinical features may appear at different times throughout childhood followed by progressive multiorgan failure which reduces life expectancy where patients rarely live beyond their 5th decade of life (2). Although clinical assessment is considered as the first line of diagnosis, gradual multiorgan dysfunction and variable expressivity of the disease within the same family members make it more challenging (3). Clinical management of AS is also complicated as patients present a combination of endocrine, cardiac, renal, neural, and hepatic defects (4). At present, there is no specific therapy for AS; however, early diagnosis and symptom-targeted treatment can prevent or slow down the progression of the disease as well as improve the life span (5).

The genome-wide linkage and homozygosity mapping study in a large French kindred has demonstrated that molecular defects in the *ALMS1* gene localized on Chr 2p13.1 are causal mutations (6). ALMS1 gene encompasses 23 exons and codes for a 461-kDa protein with 4,169 amino acids. ALMS1 protein shows ubiquitous expression and is located at centrosomes and basal bodies of ciliated cells, suggesting its probable role in the maintenance of cytoskeleton, regulation of cell division, intraciliary transport, cellular migration, and endosomal recycling (7). At present, ~330 disease-causing mutations (166 missense, 8 splice site, 156 indel) in the ALMS1 gene have been reported in mutation databases. Among the missense mutations, ~90% were observed in exons 8, 10, and 16, making them “mutational hotspot” regions of the ALMS1 gene.

AS is reported in all major ethnic populations like Caucasians, Asians, Africans, Americans, and Arabs (8–10). However, majority of the published studies on AS are conducted in patients belonging to non-Arab ethnicities. Owing to the distinct cultural traditions followed in Arab countries, we believe that studying AS patients from Saudi Arabia might present interesting clinical and genetic findings. Saudi Arabia is regarded as the epicenter of consanguinity with more than 50% marriages (close relatives among extended families). Consanguinity is known to enrich the prevalence of defective alleles in the population and is the predominant reason for high incidence of genetic diseases in certain countries such as Saudi Arabia and Pakistan (11, 12). So far, the number of studies reporting on sporadic AS cases is greater than on familial forms. Hence, familial cases have great potential to characterize the genetic basis of the disease by identifying novel mutations in *ALMS1*, deducing mode of inheritance, genotype–phenotype correlation, and distinct clinical phenotype presentations. In this study, we aim to use whole-exome sequencing (WES) technology on two unrelated Saudi families to simultaneously characterize the molecular basis of AS and identify casual genetic variants. We also employed various system biology approaches to explore the consequences of ALMS1 pathogenic mutation on the gene function.

## Materials and Methods

### Patients Recruitment and Clinical Assessment

This study is conducted upon the approval of ethics committees from Al-Hada Armed forces hospital, and King Abdulaziz University Hospital, Jeddah. Initially, AS patients were recruited from the pediatric hepatology and pediatric cardiology clinics, in Al-Hada Armed Forces Hospital, Saudi Arabia. Clinical examination of multiple organs among AS patients were conducted using combined investigations such as electrocardiogram, radiological tests (x-ray), ultrasonography of abdomen, audiometry, and visual evoked potential (13). The extensive family data of the patients were collected by careful interviews by genetic counselors. These families were subsequently referred to genetic medicine clinic for genetic testing, diagnosis, and counseling. Written informed consent was also obtained from all participants of both families.

### DNA Isolation

Using a sterile EDTA tube, 2–3 ml of venous blood was collected from each participant and stored at −20°C. Total genomic DNA was isolated using a commercial kit as per the manufacturer's instructions (Wizard^®^ Genomic DNA Purification Kit, Promega). DNA concentration and quality (260:280 ratios) were measured at 260 nm using a NanoDrop spectrophotometer. The integrity of genomic DNA was confirmed using agarose gel electrophoresis as part of qualitative assessment.

### Whole-Exome Sequencing and Variants Filtering

One hundred microliters (55 ng/μl) of high-quality genomic DNA (260/280 ratio is 1.9) was used for WES. Library preparation was performed using “Sure Select QXT All human exon V6 (Agilent)” to prepare 60 Mb exome library followed by sequencing on the HiSeq 2000 Next Generation Sequencer platform. Sequencing reads were aligned against human genome reference assembly build 38 (GRCH38.p12) with the help of BLAST (version 0.6.4d). Base quality was recalibrated using the GATK tool (GATK; www.broadinstitute.org/gatk). The alignment of sequencing data revealed 100× coverage for up to 75% of the target region. SAM tools were used for base calling to identify single-nucleotide polymorphisms (SNPs) and indels (14). Variant prioritization and filtering steps were conducted as per the following criteria: a minimum Phred score of 40 (base quality), rare allele frequency (MAF < 0.01), functional variants (coding or regulatory regions), pathogenicity based on variant prediction tools (SIFT, Polyphen, and CADD), and allelic zygosity (homozygous or compound heterozygous in patients).

### Sanger Sequencing Validation of *ALMS1* Mutation

Potential *ALMS1* candidate mutation in affected family members and population healthy controls was validated using the Sanger sequencing method. Primer sequences targeting an average amplicon size of 400–500 bp (forward primer: 5′-GTCTTTCTAACTTGGGATCAGAG-3′ and reverse primer: 5′-CCTCCAGGGTCTGGTCTTG-3′), were designed using the NCBI-primer BLAST online program, followed by PCR and bidirectional dideoxynucleotide sequencing reactions. The BioEdit program was used for sequence alignment and annotation of nucleotide sequence mismatches (http://www.mbio.ncsu.edu/). All *ALMS1* mutations were annotated against the ENST00000613296.4 reference m-RNA sequence. The position of mutation was determined according to human genome variation society guidelines, which recommended to consider “A” of ATG codon as the 1st nucleotide in the mRNA.

### Computational Biology Analysis of *ALMS1* Mutation

Computational analysis of potential *ALMS1* mutation was performed based on the results obtained from WES and Sanger sequencing. Our system biology approach was subjective to the nature of *ALMS1* mutation and its effect on RNA or protein sequence. We have initially used Human Splicing Finder (HSF), a publicly available web server (http://www.umd.be/HSF/) to predict the impact of mutations on the strength of 5'ss, 3'ss, and branch splicing points. For predicting the impact of the mutation on *ALMS1* RNA secondary structure, we used RNAfold web server (http://rna.tbi.univie.ac.at/cgi-bin/RNAWebSuite/RNAfold.cgi) (29), which uses the loop-based energy model and the dynamic programming algorithm for prediction of the linear or circular single-stranded RNA structure (16). The minimum free energy (MFE) value differences were compared to measure mutation-induced stability differences between wild-type and mutant secondary structures of *ALMS1* and RNA molecules.

## Results

### Identification of the *ALMS1* Variant in AS Patients

In this study, we present the whole-exome sequencing results of six AS patients (three from each family) belonging to two unrelated families from Saudi Arabia. In both families, parents were first cousin-consanguineous marriages. Exome sequencing of each AS patient has yielded an average number of 6.9 billion bp read bases and 46 million bases, with 51.2% of GC content and 93.2% of Q30. The average read length was 148.96 base pairs, and the cumulative depth distribution value in the target region for >60% of bases was 50× or greater. Post quality control filtering, we found approximately 96.5% of known nucleotide variants, previously reported in public databases. From each exome, approximately 96,000 SNPs, including 12,153 synonymous variants, 12,000 missense variants, and 11,500 indels were identified.

Since ALMS1 pathogenic mutations are known to be clustered around hotspot regions, we have initially screened biallelic mutations located in exons 5, 8, 10, 11, 16, 17, and 18. Interestingly, the mutation search of all AS patients has not revealed the presence of either coding or regulatory region mutations in the hotspot regions of ALMS1 gene. However, interestingly, we found a novel ALSM1 mutation (c.11873–2A>T) at the intron 18–Exon-19 boundary, which is not reported in any of the public resource genetic databases like gnomAD,1000 genomes, ExAC, dbSNP-NCBI, and Saudi Human Genome Project (SGHP). Table 1 summarizes the genotype–phenotype correlations for all splice site mutations. The CADD score of this mutation is 24.5, which classifies it as likely pathogenic. Since all the six AS patients carried this mutation, we assumed that all the patients have inherited one copy of the defective allele from each parent. Then, the validation of exome sequencing derived c.11873–2A>T mutation in all the probands and other family members were carried out by Sanger sequencing, which confirmed the absence of this mutation in homozygous form among the remaining family members. None of the 100 healthy controls had this pathogenic mutation, whether hetero- or homozygous states.

**Table 1:** Genotype–phenotype correlations for ALMS1 splice site mutations in Alström syndrome patients.

**References**	**(17)**	**(18)**	**(19)**	**(****20****)**		**Current study**					
						**Family A**			**Family B**		
				**Patient 1**	**Patient 2**	**II.6**	**II.7**	**II.8**	**B: II.4**	**B: II.5**	**B: II.6**
**Clinical symptoms**	**c.11550+3A>T**	**c.11672–2A>G**	**c.11876–3T>G**	**c.11876–2A>T**		**c.11873–2A>T**			**c.11873–2A>T**		
Photophobia		+				+	+	+	+	+	+
Retinal dystrophy	+	+	+	+	+	+	+	+			
Vision loss	+	+	+	+	+	+	+	+			
Nystagmus	+	+		+	+	+	+		+	+	+
Obesity	+	+				+	+	+			
Dilated cardiomyopathy	+	+				+	+				
Hearing loss	+		+			+	+	-			
Insulin resistance	+		+	+	+						
Short stature	+			+	+				+		
Diabetes insipidus			+			+	+	-			
Epilepsy			+								
Hypogonadotropic hypogonadism			+						+	+	+
Acanthosis nigricans	+	+		+	+	+	+	+	+		+
Hypothyroidism									+	+	+

*Patients 1 and 2 are mentioned as patients 4 and 5, in the original article as Saudi Arabs (20). “+” reflects presence of the symptom, whereas no marks mean absence or no data for the concerned symptom available*.

### Inheritance Mode of *ALMS1* Mutation

#### Family A

**Figure 1:**
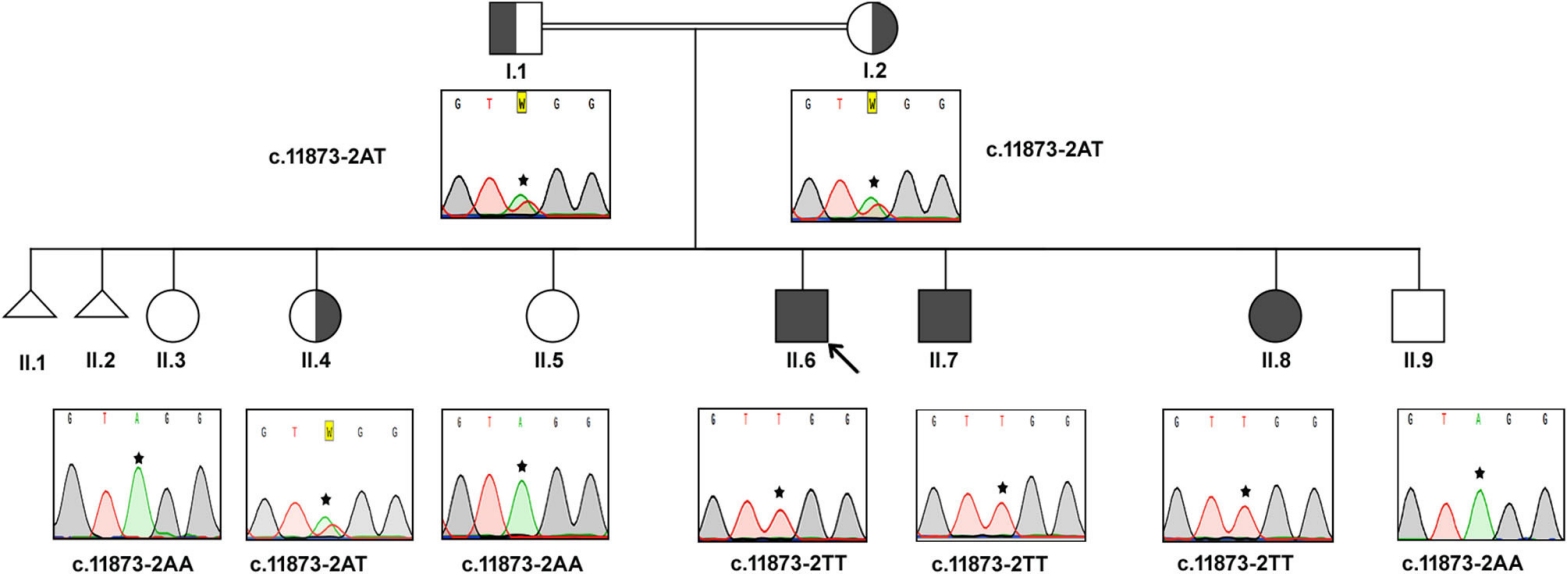
Pedigree and the DNA sequence analysis of AS Family A. Proband. (II.6) is indicated by the arrow. Each DNA chromatogram (reverse strand) shows the result from the respective Sanger sequencing confirming the status of the mutation. The proband is having the homozygous TT mutation at the splice site (c.11873–2). Both parents are heterozygous for the given mutation. All three affected siblings show homozygous mutations while the unaffected siblings were either heterozygous for the mutation or homozygous for the normal allele.

In this consanguineous family, both father (I.1) and mother (I.2) were heterozygous carriers of c.11873–2 A>T mutation (Figure 1). The index case (II.6, 17 years) and the two siblings (II.7, 15 years & II.8, 8 years) were homozygous to the T allele. Among other siblings, three sisters (II.3, II.4, II.5) and one brother (II.9) were homozygous to the wild-type allele “A.” The mode of inheritance of *ALMS1* mutation confirms that both defective alleles were transmitted from parents to AS patients in an autosomal recessive pattern. All the three affected patients including the proband (IV.6, IV.7, IV.8) showed common clinical symptoms including severe photophobia, progressive visual loss, obesity, bilateral horizontal nystagmus, and flat feet. Diabetes mellitus, cardiomyopathy, and mental disability were observed in probands IV.6 and IV.7 only.

Proband II.6 is severely photophobic and experienced a progressive loss of visual acuity since the age of 9. His eyes showed bilateral horizontal nystagmus, and hearing loss since the age of 11. II.6 has also manifested hepatic dysfunction, sensorineural hearing loss (SNHL), speech impairment, and acanthosis nigricans. He reported mild continuous palpitations and was NYHA class III. He did not have recurring infections but had a history of balancing problems in gait. II.7 presented with progressive visual acuity loss since the age of 3. He was severely photophobic, like his elder brother (II.6). He had an acquired bilateral horizontal nystagmus. He only complained of tinnitus, not hearing loss. He has also complained of episodes of profuse epistaxis, sometimes during sleep. He manifested with acanthosis nigricans and was on oral hypoglycemic. His frequency of urination increased, and he had nocturia. He reported the sensation of pounding continuous palpitations. He was NYHA class II and reported general fatigue. Both II.6 and II.7 had a history of long standing cough with yellow sputum that is precipitated by moderate activity.

Patient II-8 presented with photophobia and visual loss since a very young age (<5). She did not complain of hearing loss or tinnitus. She manifested with acanthosis nigricans and had a history of persistent weight gain and increased appetite. She did not have urinary habit changes or nocturia. She had dental abnormalities and was diagnosed with hypertension. She had mood swings and numbness on her left hand and foot. Their clinical follow-up revealed that none of them have developed renal failure or recurrent infections or malignancies. This family reported thyroid pathology symptoms consistent with hypothyroidism (aversion to cold, dryness, and myxedema).

#### Family B

**Figure 2:**
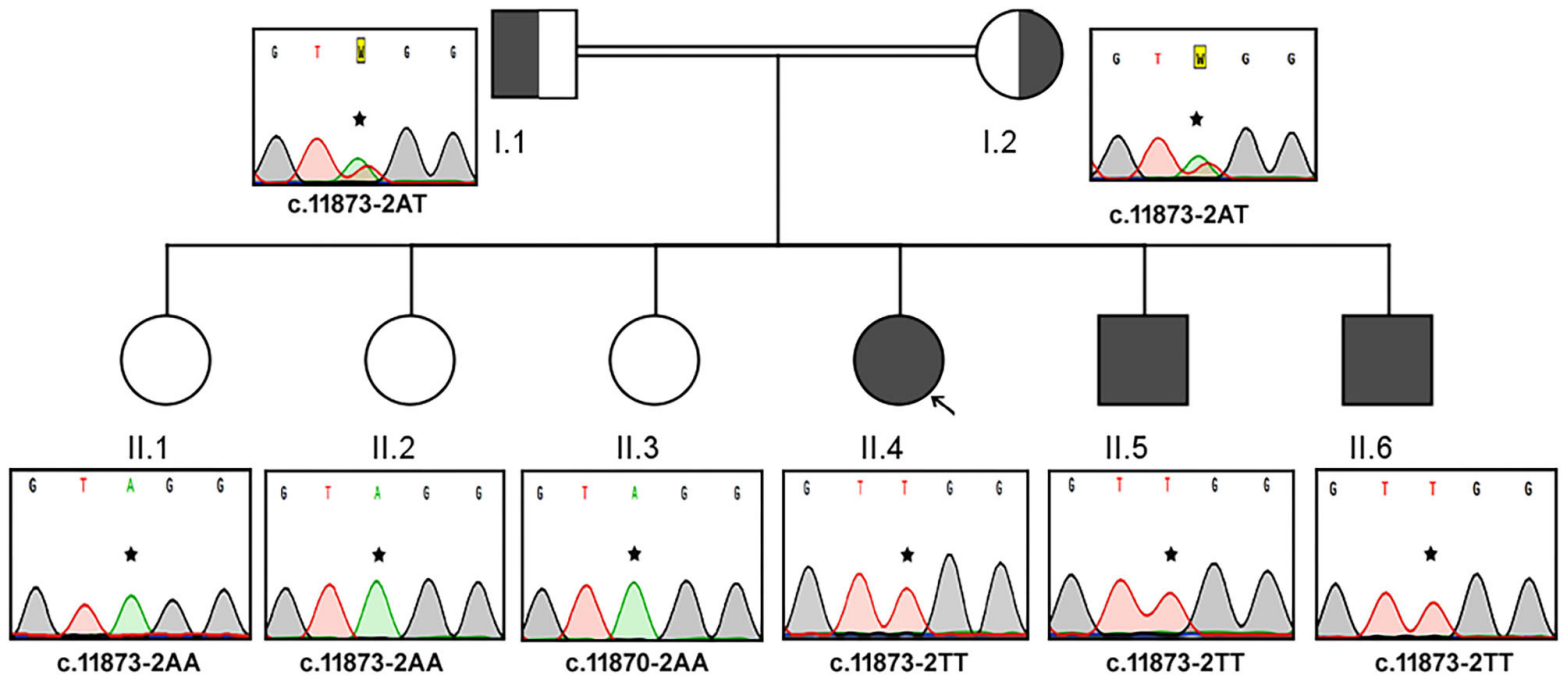
Pedigree and the DNA sequence analysis of AS Family B. Each DNA chromatogram (reverse strand) shows the result from the Sanger sequencing confirming the status of the mutation. Both parents (I.1 and I.2) are heterozygotes, three AS patients are homozygous for TT, while the three unaffected siblings are homozygotes for AA genotype for the given genetic location.

The Sanger sequencing findings, after the exome sequencing identified the mutation, of this consanguineous AS family are as follows; the proband (II.4) and her two brothers (II.5 and II.6) were homozygous to T minor allele, whereas their healthy siblings were homozygous to A major allele (II.1, II.2, and II.3) and both parents (I.1 and I.2) were heterozygous (AT) for ALMS1, c.11873–2A>T mutation. These results confirm that the AS patients in this family have inherited one copy of defective T allele from each parent, confirming the autosomal recessive mode of inheritance (Figure 2). The probands II.4 (12 years), II.5 (9 years), and II.7 (4 years) showed initial symptoms of vision dysfunction (severe progressive retinal dystrophy and cataract) and dilated cardiomyopathy. The common eye manifestations are photophobia, nystagmus, and squint easily identified by parents early between 3 and 36 months. All the three patients had visual manifestations before the first 36 months of their life. The proband (11.4; 12 years) had reported photophobia since her first year. She had bilateral horizontal nystagmus, progressive loss of visual acuity, and bilateral sensorineural hearing loss. She used to sleep during the day and mostly avoided daytime because of her photophobia. She was obese (>95 percentile) with polyphagia and acanthosis nigricans. At the age of 5, she was diagnosed with autism spectrum disorder. She was short for her age and had microcephaly but did not have any extra or fused digits. She was also diagnosed with hypothyroidism. She did not have any allergies. Probands II.5 and II.6 presented SNHL and endocrine-related disorders like impaired glucose homeostasis, hypothyroidism, hypogonadism evidenced by small testes, and precocious puberty. Acanthosis nigricans and sparse hair were also observed in both of them. The remaining three sisters (II.1, II.2, and II.3) were healthy and did not present any symptoms of the disease. Both parents were apparently healthy and mention no significant health complications related to Alström Syndrome (I.1 and I.2).

### Computational Analysis of *ALMS1* Mutation

#### Splice Site Prediction

The HSF analysis of *ALMS1* variant (c.11873–2A>T) in the splice site (tgtttcctgt*AG*ga >> tgtttcctgt*TG*ga) predicted the consensus values (CV) of 87.32 and 59.45 for the wild-type active sites and mutant active sites, respectively. The CV variation score of −31.92% between wild-type and mutant alleles suggests that the loss of wild-type splice site acceptor (*A*G>*T*G) would result in the skipping of exon 19 and instead accept the splice site acceptor sequence (AG) of exon 20 of *ALMS1*.

#### RNA Secondary Structure Analysis

**Figure 3:**
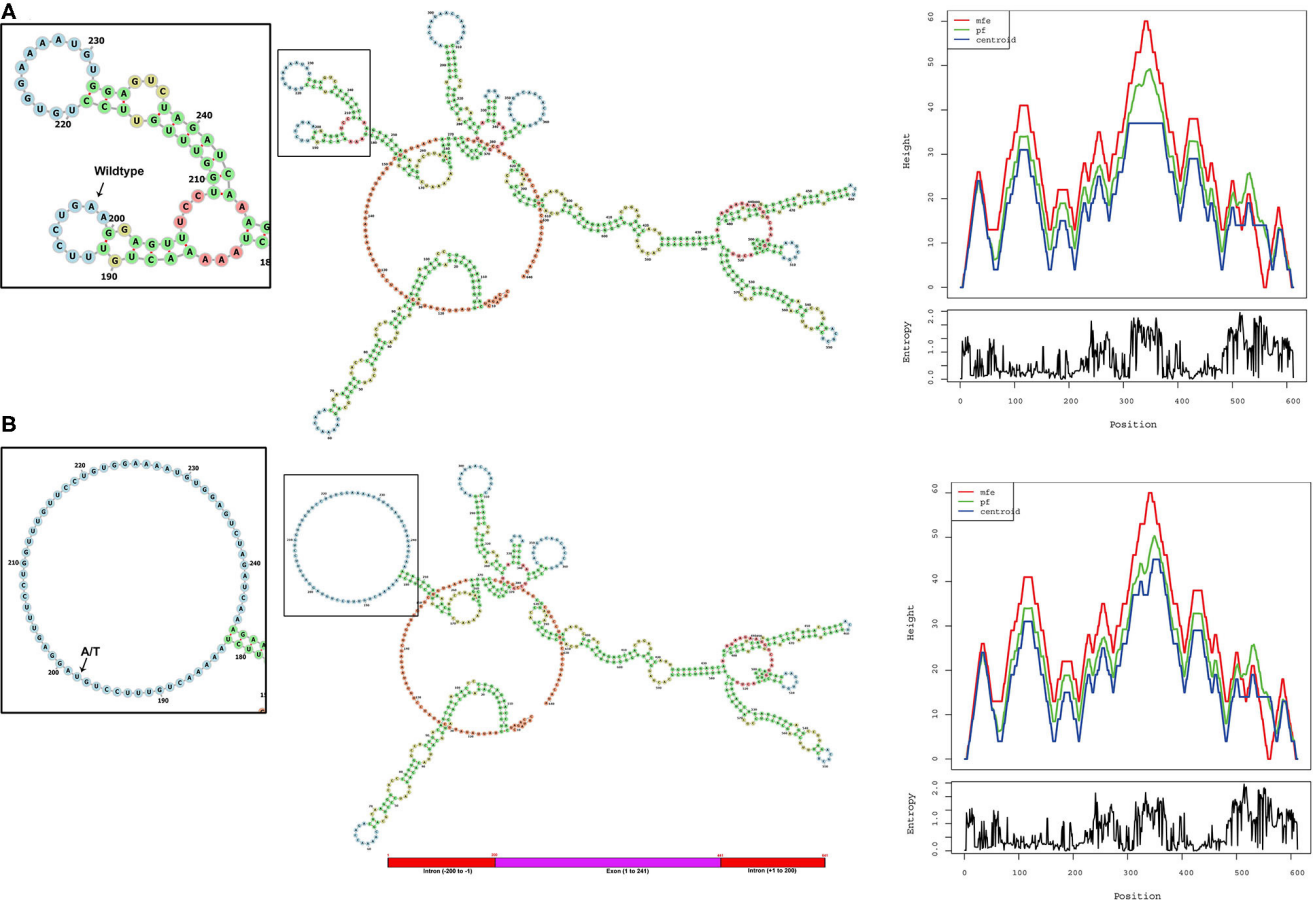
Computational analysis and the RNA secondary structure predictions. The secondary structure predictions of **(A)** native and **(B)** variant RNA structures of ALMS1 using RNAfold software. Inset shows the enlarged picture of the region containing the native or the variant part of the RNA. Base-pair probability is indicated in the key. Mountain plot shows the secondary structures in a height vs. position, where the helices are represented in slopes, loops in plateaus, and hairpin loops in the peaks.

The *ALMS1*, RNA secondary structure is characterized by helix, loops, stem, and dangling ends. Although both wild-type and mutant RNAs showed structural similarity, their centroid structures revealed differences in their minimum free energy (MFE) values. In the native state, A nucleotide at c.11873–2, located in the hairpin loop, confers stability (144 K.Cal/Mol of free energy) to the secondary structure of *ALMS1*, RNA compared to the variant state (c.11873–2 A>T), where both sizes of the hairpin loop is seen enlarged and free energy is decreased (−175.8 K.Cal/Mol of free energy). Hence, it is assumed that the lower stability of mRNA with c.11873–2 A>T is likely to affect the mRNA folding pattern and tertiary structure formation (Figure 3).

## Discussion

Homozygous or compound heterozygous mutations in the ALMS1 gene results in AS disorder. Hence, early identification of defective variants of ALMS1 in potential asymptomatic child and carriers will provide better clinical management and options for prenatal diagnosis, respectively. Whole-exome sequencing offers an efficient and cost-effective approach in identifying *ALMS1* mutations with greater depth. Although whole-exome sequencing generates the unbiased exonic variant data from all coding genes, variant screening strategy first targets exons 8, 10, and 16 regions of the ALMS1 gene, which can solve the genetic basis in almost 90% of cases (1). However, the lack of ALMS1 exonic mutations in some AS patients suggests the possibility for the regulatory region mutations. The genetic and clinical data of Alström Syndrome of non-Arab patients is relatively well published compared to Arab patients, who inherently possess large blocks of homozygosity due to their traditional consanguineous marriage practices (8, 9, 18, 21–24). Many rare or novel mutations in different autosomal recessive diseases are widely reported in Saudi Arabian populations (25, 26).

There are few publications which reported the genetic basis of AS patients from Saudi Arabia. Table 2 summarizes the ALMS1 mutations and clinical phenotypes of Saudi AS patients. The first study conducted on four Saudi AS patients used genetic linkage mapping and Sanger sequencing and reported allelic heterogeneity of ALMS1 mutations, i.e., c.5534 C>G (S908X) and c.5981delCAGA(1992X) mutations in exon 8, c.8275C >T (R2720X) mutation in exon10, IVS18-2 A>T in exon 18 (20). Whole-exome sequencing of a 10-year-old Saudi girl presenting with diabetic ketoacidosis, hearing loss, and blindness found a homozygous frameshift deletion in exon 20 (c.12154_12166del) which creates premature termination codon (p.Arg4052Glyfs*2) in ALMS1 gene (27). Another whole-exome sequencing study on a 5-year-old Saudi girl, with photophobia, marked nystagmus, and retinal changes with short fingers tapering born to the consanguineous marriage, has reported a pathogenic mutation in ALMS1 gene (c. 8441C>A, p.S2814^*^) (31). By using whole-exome sequencing, earlier our group found causative rare biallelic mutations in the ALMS1 gene in exon 8 (T376S in exon 5, and S909^*^) and exon 10 (R2721^*^) among AS patients from two other unrelated Saudi families (28). These truncating mutations at residues S909 and R2721 possibly create an unstable protein due to loss of CC domain and ALMS motif on the C-terminal end which led to intracellular truncated protein degradation (31). Another retrospective study from Saudi Arabia has reported the identification of 10 ALMS1 different mutations (E3649^*^, Q2648^*^, p.E913Sfs^*^20, p.Ser2102^*^, p.Arg2928^*^, p.Ser2102^*^, p.Arg2722^*^, p.P3911QfsX16, p.Ser908^*^, IVS18-3A>T) in 19 Alström cases presenting different ophthalmic phenotypes from 13 Saudi families. Their finding underscored the point that visual phenotypes in children should raise suspicion for Alström syndrome (30).

**Table 2:** Summary of genetic mutation vs. clinical features observed in Saudi Alström patients.

**Variant**	**Exon**	**Impact on protein**	**Age and sex**	**Clinical expression**											**References**
				**O**	**SS**	**CN**	**PP**	**DCM**	**LoV**	**CRD**	**MR**	**IR**	**SNHL**	**HD**	
c.5534 C>G	8/23	S908X	8, M	+		+	+		+	+					(27)
c.5981 delCAGA	8/23	KL1992X	2, M		+	+	+	+	+	+					
c.8275 C>T	10/23	R2720X	7, M			+		+	+	+					
IVS18-2 A > T	19/23		10, F	+					+	+					
″	″	″	6, M	+	+				+			+			
c.12154_12166del	20/23	p.Arg4052Glyfs^*^2	10, F		+				+	+	+	+	+		(27)
c.8441C>A	10/23	p.S2814^*^	5, F			+	+		+			+	+		(28)
c.2938dupA	8/23	p.M980Nfs^*^9	21, M	+				+	+				+		(29)
	”	”	16, M					+	+				–		
c.1159 A>T	5/23	T376S	III.2	+	+	+	+	+	+		+		+		(30)
c.2759 C>G	8/23	S909^*^													
	”	”	III.4	+	+			+			+		+		
c.8194 C>T	10/23	R2721^*^	14, F	+		+		+	+		+		+		
		”	12, M	+				+	+				+		
		”	9, M	+		+		+	+		+		+		
c.7942C>T	10/23	p. Q2648^*^	2, M	+					+	+					(19)
c.2737_2740delGAGA	8/23	p.E913Sfs^*^20	2, M	+					+						
c.6305C>A	8/23	p.Ser2102^*^	2, M							+					
c.11876–2A>T	18/23	–	2, M						+		+		+	+	
c.8782C>T	10/23	p.Arg2928^*^	3, F	+				+	+		+				
c.6305C>A	8/23	p.Ser2102^*^	3, F	+					+		+				
c.8164C>T	10/23	p.Arg2722^*^	3, M	–				+		+					
c.11732delC	18/23	p.P3911QfsX16	3, M	+						+					
c.5534C>G	8/23	p.Ser908^*^	6, M	+						+					
c.11876–2A>T	18/23	–	8, F	+				+			+				

*O, obesity; SS, short stature; CN, congenital nystagmus; PP, photophobia; DCM, dilated cardiomyopathy; LoV, loss of vision; CRD, cone rod dysfunction; MR, mental retardation; IR, insulin resistant; SNHL, sensorineural hearing loss HD, hepatic disease. “+” marks presence of the symptom; whereas no marks mean absence or no data of the concerned symptom available*.

There are six ALMS1 splice site mutations (c.454–5T>G, c.7677+1G>T, c.10388–2A>G, c.11550+3A>T, c.11672–2A>G, c.11876–3T>G, c.11876–2A>T) reported in previous publications (7, 17–20, 30). In the current investigation, through whole-exome sequencing of AS patients from the consanguineous background, we observed a rare homozygous mutation (c.11873–2A>T) at the acceptor splice site of exon 19 of the ALMS1 gene. This mutation is similar to the previous publications which reported it in sporadic cases (19, 20, 30). From its autosomal recessive mode of inheritance pattern of ALMS1 mutation and subsequent computational functional analysis, we propose that this mutation is most likely disease causative in these families. Despite the lack of overlap between the lineages of the two studied families, we assume that c.11873–2A is possibly a founder mutation in Saudi Arabia. In eukaryotes, dinucleotides at the 5′ (GT) and 3′ (AG) ends of intronic sequences are recognized as exon boundaries, which are subsequently acted by the spliceosome complex to remove introns (32). The A>T transversion converts c.11873–2 the 3′ AG splice acceptor sequence to TG, due to which the RNA spliceosome complex skips the entire exon 19 (Chr2: 73,601,195–73,601,436) and accepts the next 3′ AG splice acceptor of exon 20, resulting in a shorter version of ALMS1 mRNA by 241 nucleotides.

In the absence of functional data, we tried to understand the putative functional role of the c.11873–2A variant on the secondary structural features of ALMS1 mRNA using RNA fold software, which predicted a free energy difference of −31 K.Cal/Mol between native and variant forms. Hence, it is assumed that the lower stability of mRNA with c.11873–2 A>T is likely to affect the mRNA folding pattern and tertiary structure formation. The change in the secondary structure of the splice site variant RNA may further alter its function which could primarily occur via the two possible mechanisms. Firstly, in the absence of the correct folding of the RNA, the physiological functioning of the RNA which is mediated through the potential interaction of RNA with the protein complexes like spliceosome complex (33) would be lost or change and thus affect proper RNA transport or mRNA splicing or both. Secondly, due to the improper folding of RNA, potential binding sites of micro-RNA (miRNA) could be lost or modified, leading to an altered cellular condition that may lead to the disease state (32, 34).

The mechanistic link between ALMS1 genotype vs. AS protein phenotype is not yet well-understood, partly because of limited functional data exploring the role of catalytic domains in protein function. Of the 4,166 aa long native ALMS1 protein, exon 19 encodes 80 amino acids (3,958–4,038 aa) lying in between serine-rich (3,857–3,873 aa) and ALMS1 motif regions (4,037–4,166 aa) at the c-terminal region of ALMS1 protein. It is assumed that exon 19 skipping by the c.11873–2A>T variant results in a short polypeptide (4086 aa), which could affect the loss of secondary (α-helices, and β-pleated sheets) and tertiary (3D) and quaternary (biomolecular complexes) structural features, in addition to biological functions (microtubule organization, intracellular transport, endosome recycling, and cell-cycle regulation) of ALMS1 protein. It remains plausible that the primary cilium or basal body dysfunction due to defective ALMS1 among homozygous carriers contributes to many aspects of the AS phenotype including obesity, retinal dystrophy, hearing loss, kidney dysfunction, neurological disturbances, and fibrosis (1).

In conclusion, this study confirmed the autosomal recessive inheritance of a very rare *ALMS1* mutation among six AS patients from two unrelated consanguineous Saudi families. This mutation abolishes the 3′-AT splice site acceptor sequence of exon 19 due to which the entire exon 19 is skipped, shortening the mRNA by 241 nucleotides. Bioinformatics prediction tools demonstrated that this mutation destabilizes the secondary structural features of mature mRNA, hence disrupting the biological functions of ALMS1 protein. Future functional studies at the level of cDNA and protein could provide more insights about variant pathogenicity and also help to understand how this ALMS1 genotype contributes to AS clinical phenotypes. This study confirms the important utility of WES in detecting pathogenic variants occurring in either coding or regulatory regions among AS patients. Multidimensional molecular characterization of complex rare disorders is challenging owing to the variable clinical expressivity among AS patients. However, WES proved to be a valuable tool in helping clinicians in diagnosis, providing better clinical management and intervention for AS patents and their families.

## Data Availability Statement

The datasets presented in this article are not readily available because(a) participants' refusal to store or distribute the genomic data in the public domain and (b) as per the local Institutional Ethics committee approval and national policy on genomic data sharing in the public domain outside the country. Requests to access the datasets should be directed to RE, OS, or NS.

## Ethics Statement

The studies involving human participants were reviewed and approved by the ethics committee of King Abdulaziz University, Saudi Arabia. Informed consent was obtained from each tested individual prior to genetic testing. Written informed consent to participate in this study was provided by the participants' legal guardian/next of kin.

## Author Contributions

OS, BB, NK, RE, and NS: conceptualization. NS and BB: data curation. RE: formal analysis. NA: funding acquisition and project administration. AS, HA, AM, NS, and BB: methodology. BB: software and visualization. OS, JA-A, NS, and RE: supervision. BB and KN: validation. OS, BB, NK, AA, JA-A, NS, and RE: writing of original draft and editing. All authors contributed to the article and approved the submitted version.

## Conflict of Interest

The authors declare that the research was conducted in the absence of any commercial or financial relationships that could be construed as a potential conflict of interest.
